# The Association of Serum Thrombomodulin with Endothelial Injuring Factors in Abdominal Aortic Aneurysm

**DOI:** 10.1155/2017/2791082

**Published:** 2017-04-03

**Authors:** Magdalena Budzyń, Bogna Gryszczyńska, Wacław Majewski, Zbigniew Krasiński, Magdalena Paulina Kasprzak, Dorota Formanowicz, Krzysztof Wojciech Strzyżewski, Maria Iskra

**Affiliations:** ^1^Department of General Chemistry, Chair of Chemistry and Clinical Biochemistry, Poznań University of Medical Sciences, Poznań, Poland; ^2^University Hospital of Lord's Transfiguration, General and Vascular Surgery Clinic, Poznań, Poland; ^3^Department of General and Vascular Surgery, Poznań University of Medical Sciences, Poznań, Poland; ^4^Department of Clinical Biochemistry and Laboratory Medicine, Poznań University of Medical Sciences, Poznań, Poland

## Abstract

*Background.* The aim of the present study was to evaluate the concentration of serum thrombomodulin (sTM) in the AAA patients and to examine its correlation with various factors which may potentially participate in the endothelial injury.* Materials and Methods.* Forty-one patients with AAA were involved and divided into subgroups based on different criteria. Concentration of sTM was measured using enzyme-linked-immunosorbent assay (ELISA). The results were compared with those obtained in 30 healthy age- and sex-matched volunteers.* Results.* The higher concentration of sTM was observed in AAA patients compared with those in controls volunteers [2.37 (1.97–2.82) ng/mL versus 3.93 (2.43–9.20) ng/mL,* P* < 0.001]. An elevated sTM associated significantly with increased triglycerides (TAG) [*P* = 0.022], cholesterol [*P* = 0.029], hsCRP [*P* = 0.031], and advanced glycation end products (AGEs) [*P* = 0.033].* Conclusions.* The elevation of serum sTM level suggests that endothelial damage occurs in AAA pathogenesis. The correlations observed indicate that lipids abnormalities, inflammation, and oxidative stress may be involved in this destructive process.

## 1. Introduction

Abdominal aortic aneurysm (AAA) is a common degenerative disease characterized by thinning of aortic media with refraction of vascular smooth muscle cells and extracellular matrix destruction [1, 2]. The inflammatory and degenerative changes of the aortic wall lead to a decreased tensile strength and progressive dilation, ultimately resulting in AAA rupture. An overall mortality for this illness approaches 90% and the only way to prevent a patient's death is the elective open surgical or endovascular aneurysm repair [3]. However, both progressive disease and invasive surgical interventions carry a significant risk. For this reason the mechanisms involved in the pathogenesis of AAA have been extensively studied to find new strategies in the AAA diagnosis and treatment.

 Some findings demonstrate that endothelial cell injuries may represent a basic step in the process of aneurysm formation [4–6]. A loss of the endothelial layer integrity may trigger the infiltration of immune cells, stimulate intraluminal thrombus formation, and affect smooth muscle cell proliferation and migration. Endothelial cells undergoing injury release a variety of soluble particles known as markers of endothelial damage [7–9]. The circulating endothelial particles can be quantified and may be promising candidates for clinical testing. One of them is a soluble serum thrombomodulin (sTM), an endothelial bound protein [10–12], whose level was found to be increased in conditions associated with vascular risk such as peripheral and coronary atherosclerosis disease, diabetes mellitus, rheumatoid arthritis, and systemic lupus erythematosus [12–17].

The concentration of markers of endothelial damage in the pathogenesis of AAA is largely unknown. There are only two minor studies investigating the association of a circulating thrombomodulin with AAA which reported contradictory findings [18, 19]. There are also a lot of doubts with regard to the mechanisms involved in endothelial vascular injury in AAA pathogenesis. The inflammatory response, increased metalloproteinase's activities, and oxidative stress are supposed to participate in this destructive process [20–22]. Since it is well documented that aneurysmal disease shares many risk factors with cardiovascular diseases, much effort has been devoted to determine the effect of these factors on aortic dilation in AAA development [23–25]. Although lipid abnormalities are known as a major cause of injury to vascular endothelium in atherosclerosis [26, 27], their role in AAA formation has not been fully understood.

Therefore, the aim of the present study was to evaluate sTM concentration in patients undergoing a surgery for the repair of an AAA and examine its association with the disease severity reflected by aneurysm size. The second purpose was to evaluate the correlation of sTM with factors which may potentially participate in the endothelial injury, with a special attention focused on inflammation, lipid, and oxidative stress parameters. In the last part of our study we tried to determine the effect of two different surgical techniques of AAA repair on endothelium by making comparison between pre- and postoperative concentration of sTM in each subgroup of patients qualified to open surgery (OR) or endovascular aneurysm repair (EVAR).

## 2. Materials and Methods

### 2.1. Patients

The study was performed in a group of forty-one AAA patients (32 men and 9 women; mean age 71.82 ± 9.48), who were admitted to the Department of General and Vascular Surgery at the University of Medical Sciences, Poznań, Poland. AAA patients underwent Doppler ultrasonography, computed tomography, or arteriography before surgery. The maximal abdominal aorta diameter was measured by reviewing each coronal CT section. The indications for elective AAA surgery were based on size or growth rate and followed the recommendations of International Society for Cardiovascular Surgery (the AAA greater than 4 cm in diameter, or more than twice the diameter of the normal infrarenal and/or a six-month expansion rate of 0.5 cm or more). Cut-off values (3–5.5 cm) were used to define normal aorta, small AAA, and large AAA. AAA patients were examined to diagnose any concomitant diseases and provide proper treatment to prevent surgical complications. The preoperative data (particularly regarding cardiovascular risk factors) and concomitant diseases are summarized in Table 1. Twenty-two patients were classified as a high-surgical-risk and/or with anatomic characteristics favorable to endovascular aneurysm repair (EVAR) (Tables 2 and 3). Nineteen patients were classified as low-surgical-risk patients and submitted to conventional open AAA repair (OR).

All patients on the day of surgery were on statins but only 20 AAA patients (50%) had been receiving statins for at least 3 months before surgical treatment. Furthermore, the administration of the indicated oral medications, especially *β* blockers and ACE inhibitors, had been continued until the surgery. Preoperatively, an epidural catheter with bupivacaine was introduced and preoxygenation was performed prior to the induction of general anesthesia. Propofol and sevoflurane were administrated to all patients to maintain anesthesia. Intraoperatively, a single dose of intravenous heparin (70 U/kg of body weight) and mannitol (100 mL) was administered before clamping of the aorta or the iliac-femoral artery, respectively. Before (at 1 and 12 hours) the operation patients received a dose of an antihistamine (cetirizine hydrochloride 10 mg), and postoperatively antibiotics and nonsteroid anti-inflammatory drugs (nimesulide 100 mg twice a day) were administered for 72 hours. Venous blood samples were collected before the induction of anesthesia and 24 h, 48 h, 72 h, and 96 h after operation.

A control group of thirty volunteers (20 men and 10 women; mean age: 62.45 ± 9.23) with normal infrarenal aortic diameter were matched to AAA patients according to age, gender, and smoking habits.

### 2.2. Sample Collection

Blood samples were drawn preoperatively and postoperatively from the arms of AAA patients in the recumbent position. Samples were collected in heparin anticoagulant tubes and after 30 minutes they were centrifuged at 3.000 rpm for 15 minutes. Plasma samples were stored at temperature of −80°C until all of assays were performed. The study procedure was approved by the Bioethical Committee of the University of Medical Sciences in Poznań and informed consent was obtained from all the participants.

### 2.3. Laboratory Analysis

Hematological determination was as follows: WBC (white blood cells count), RBC (red blood cells count), and PLT (blood platelets) were made using MEDONIC M20 automatic analyzer (Clinical Diagnostic Solution, USA). Blood biochemical analysis, including the determination of total cholesterol, triglycerides (TAG), LDL-cholesterol, and HDL-cholesterol, was performed using EasyRA analyzer (Medica, USA). The sTM concentration, high sensitive C-reactive protein (hsCRP), advanced oxidation protein products (AOPP), advanced glycation end products (AGEs), soluble receptors for advanced glycation end products (sRAGE), and protein carbonyl groups were measured using enzyme-linked-immunosorbent assay (Gen-Probe Diaclone SAS, France; DRG International, USA; Cell Biolabs, USA; Cell Biolabs, USA; RayBiotech, USA; Cayman Chemical, USA; Assay Designs, USA).

### 2.4. Statistical Analysis

The statistical analysis was conducted using GraphPad Prism software 6.0 (GraphPad Software, San Diego, CA). The normality of quantitative variables was tested using the Kolmogorov-Smirnow or Shapiro-Wilk test. Any parameter not following the normal distribution was presented as median and interquartile ranges and analyzed using nonparametric Mann–Whitney test. Categorical data and proportions were compared using Chi-square or Fisher's exact test, as appropriate. Normally distributed, continuous variables were presented as a mean and standard deviation and analyzed using Student's *t*-test. Multiple group comparisons were performed by one-way analysis of variance (ANOVA) or Kruskal-Wallis test, respectively. The Pearson or the Spearman correlation coefficient was used to test the strength of any association between different variables. In all cases, *p* value ≤ 0.05 was considered significant.

## 3. Results

### 3.1. The Concentration of sTM in AAA Patients

**Figure 1:**
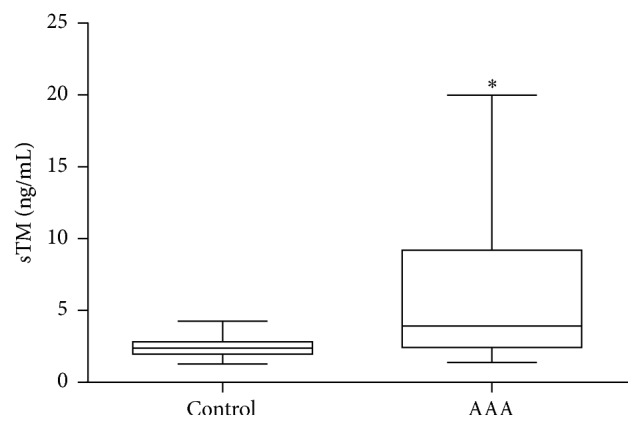
sTM concentration in AAA patients. Box and whisker plots show median (central line), upper and lower quartiles (box), and range excluding outliers (whiskers). Data were analyzed using Mann–Whitney test. ^*∗*^Statistically significant, *p* ≤ 0.05.

**Figure 2:**
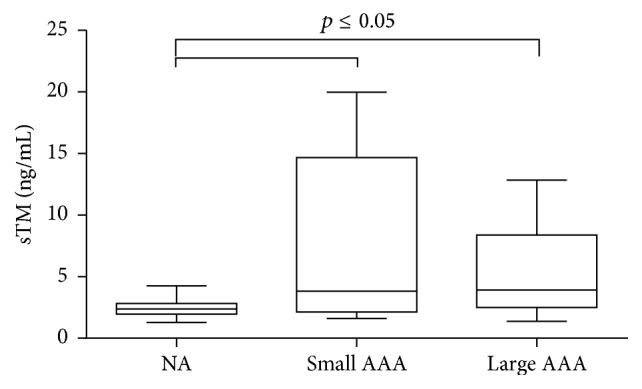
sTM concentration in AAA patients with small and large aneurysm size. Box and whisker plots show median (central line), upper and lower quartiles (box), and range excluding outliers (whiskers). Data were analyzed using Kruskal-Wallis test followed by Dunn's multiple comparison test. *p* ≤ 0.05 was considered statistically significant. NA: normal aorta.

The value of sTM concentration was significantly increased in the whole group of AAA patients, when compared with the healthy volunteers [2.37 (1.97–2.82) ng/mL versus 3.93 (2.43–9.20) ng/mL, *p* < 0.001] (Figure 1). The elevated concentration of sTM levels was noticed in patients with small [3.82 (2.14–14.68) ng/mL versus 2.37 (1.97–2.82) ng/mL, *p* = 0.032] as well as large AAA diameter [3.93 (2.51–8.38) ng/mL versus 2.37 (1.97–2.82) ng/mL, *p* < 0.001] compared with a control group with normal aorta (Figure 2). sTM levels higher than those in the controls were found in the subgroup of AAA patients with [7.93 (2.25–11.61) ng/mL versus 2.37 (1.96–2.82) ng/mL, *p* < 0.001] and without evidence of atherosclerotic disease [3.32 (2.23–5.52) ng/mL versus 2.37 (1.96–2.82) ng/mL, *p* = 0.008].

### 3.2. The Effect of OR and EVAR on sTM Concentration

**Figure 3:**
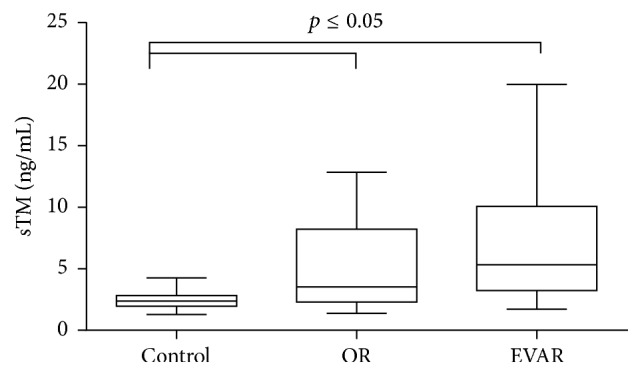
sTM concentration in AAA patients qualified for EVAR and OR. Box and whisker plots show median (central line), upper and lower quartiles (box), and range excluding outliers (whiskers). Data were analyzed using Kruskal-Wallis test followed by Dunn's multiple comparison test. *p* ≤ 0.05 was considered statistically significant.

**Figure 4:**
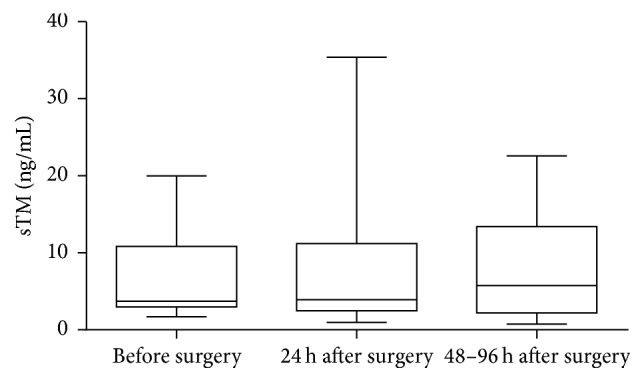
Preoperative and postoperative concentration of sTM in AAA patients who underwent EVAR. Box and whisker plots show median (central line), upper and lower quartiles (box), and range excluding outliers (whiskers). Data were analyzed using Kruskal-Wallis test followed by Dunn's multiple comparison test.

**Figure 5:**
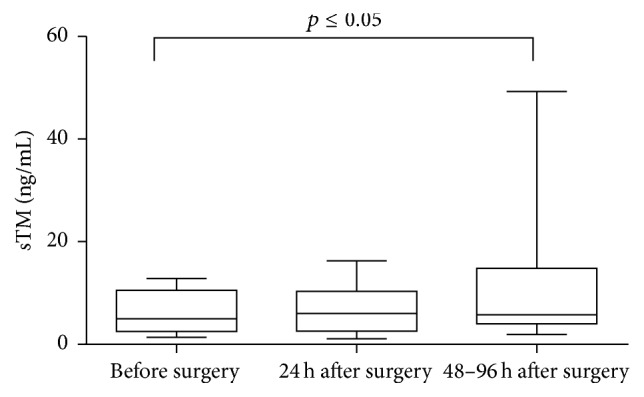
Preoperative and postoperative concentration of sTM concentration in AAA patients who underwent OR. Box and whisker plots show median (central line), upper and lower quartiles (box), and range excluding outliers (whiskers). Data were analyzed using Kruskal-Wallis test followed by Dunn's multiple comparison test. *p* ≤ 0.05 was considered statistically significant.

After qualifying patients for an appropriate surgical repair, an increased sTM concentration was observed in AAA patients submitted to OR repair [3.54 (2.30–8.21) ng/mL versus 2.37 (1.97–2.820) ng/mL, *p* = 0.015] as well as in the group qualified for EVAR [5.32 (3.25–10.08) ng/mL versus 2.37 (1.97–2.820) ng/mL, *p* < 0.001] (Figure 3). The sTM concentration demonstrated a tendency to increase in EVAR patients, but without significant difference in comparison with the rest of AAA patients [5.32 (3.25–10.08) ng/mL versus 3.54 (2.30–8.21) ng/mL] (Figure 3). The sTM concentration was measured preoperatively as well as postoperatively. The sTM level was stable in patients who had underwent EVAR. Neither in the first 24 h hours after surgery nor in longer period of time (48–96 h) was any change in sTM concentration observed [5.73 (2.22–13.42) ng/mL, 3.93 (2.49–11.23) ng/mL, versus 3.71 (3.00–10.86) ng/mL] (Figure 4). The significant rise in sTM concentration was found within 48–96 h after surgery only in patients who underwent OR [5.79 (4.01–14.81) ng/mL versus 4.99 (2.51–10.53) ng/mL, *p* = 0.013] (Figure 5).

### 3.3. Correlation of sTM with Other Parameters

**Figure 6:**
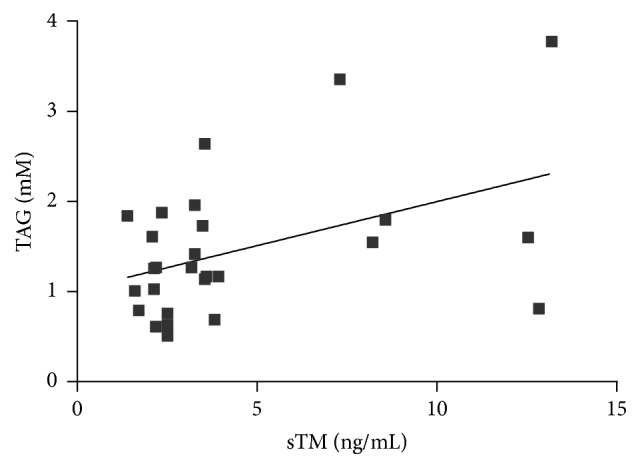
Correlation between sTM and TAG concentration in AAA patients (Spearman correlation coefficient *r* = 0.439, *p* = 0.022).

**Figure 7:**
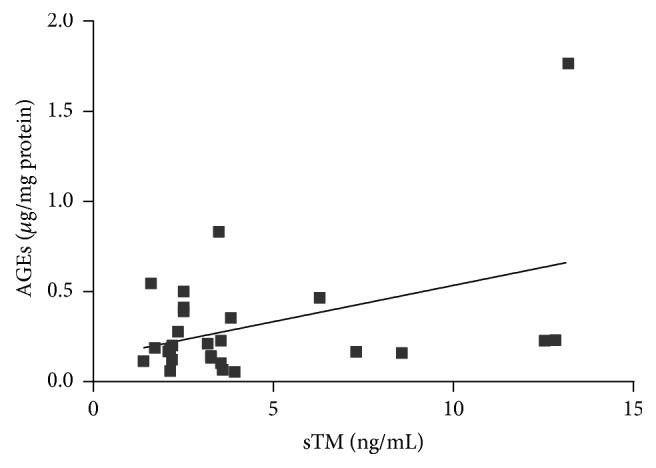
Correlation between sTM and AGEs concentration in AAA patients (Spearman correlation coefficient *r* = 0.411, *p* = 0.033).

**Figure 8:**
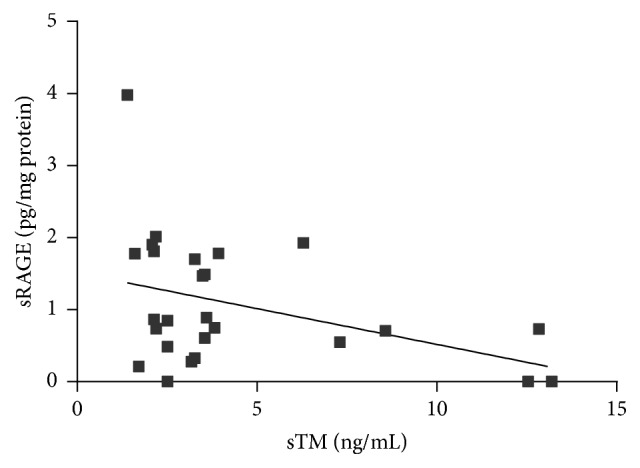
Correlation between sTM and sRAGE concentration in AAA patients (Spearman correlation coefficient *r* = −0.394, *p* = 0.046).

Among various biochemical parameters of blood, only TAG concentration had a significant positive correlation with sTM [*r* = 0.439, *p* = 0.022] (Figure 6). Taking the markers of oxidative stress into consideration, sTM was found to be significantly and positively associated with AGEs concentration [*r* = 0.411, *p* = 0.033] and negatively correlated with circulating levels of their soluble receptor, sRAGE [*r* = −0.394, *p* = 0.046] (Figures 7 and 8).

sTM concentration was analyzed as continuous and as categorical variables. All examined AAA patients were divided into 3 groups using 25th and 75th percentiles of sTM concentration distribution, respectively, as cut-off points (group I: <25th percentile, group II: 25th–75th percentile, and group III: >75th percentile).  Table 4 shows that diastolic blood pressure of AAA patients and sRAGE concentration decreased across the increasing quartiles of sTM concentration. TAG and AGEs concentration increased significantly across the increasing quartiles of sTM. Moreover, patients classified to the quartile of highest TM concentration revealed the highest cholesterol and hsCRP level (Table 4). There was no difference in either age, BMI, aneurysm diameter, hematological, or other biochemical parameters between the groups categorized according to the quartiles of sTM (Table 4).

## 4. Discussion

Endothelial cells play a major role in the maintenance of vascular integrity by controlling inflammation, thrombosis, and mural cell and matrix coverage. It is well known that the alterations of the endothelial layer participate in the development of arterial lesions associated with atherosclerosis or restenosis. The loss of endothelial integrity followed by luminal thrombus formation revealed in the histological examination of human AAA implies the importance of this process in the disease development and progression. This hypothesis was confirmed by Jamous et al., who studied the in vivo mechanisms of intracranial aneurysm formation using animal models [4]. They demonstrated that endothelial cell injury, evidenced by the loss of eNOS expression at the apical intimal pad, is the earliest pathological change in the process of aneurysm formation. Moreover, Franck et al. showed that reestablishment of the endothelial lining by cell therapy significantly inhibits AAA expansion [5]. Other authors observed that endothelial cells isolated from aortic aneurysm have altered functional properties, such as growth rate, apoptosis induction, and extracellular matrix synthesis [28].

Many endothelial products have been proposed as possible markers of endothelium damage. TM is one of the most popular indicators of endothelial injury, located on the vascular endothelium surfaces and functions as an anticoagulant. TM has an affinity for thrombin, forming a 1 : 1 thrombin-thrombomodulin complex that inhibits fibrin formation, platelet activation, and protein S inactivation [7]. Apart from the transmembrane form, TM also occurs in soluble forms in the plasma (sTM), which is probably the product of the cleaved transmembrane glycoprotein [29]. In vitro studies demonstrate that sTM is released from endothelial cells following cell membrane injury caused by the action of neutrophil derived proteases and oxygen radicals [30–32]. Elevated concentration of sTM was observed in clinical conditions associated with vascular injury such as atheromatous arterial disease, diabetes mellitus, and various vasculitis in their active phase [33–35]. In contrary to some other popular endothelial markers, such as von Willebrand factor (vWF) or tissue type plasminogen activator (t-PA), sTM does not have circadian rhythm and does not increase with age, after exercise and in an acute response to a variety of biological stimulations. For this reason plasma TM is most likely a specific marker of endothelial lesions and not of endothelial activation [7].

In our study we investigated whether the level of sTM may be a useful marker of endothelial damage in AAA. We demonstrated a significantly increased concentration of sTM in the blood of AAA patients, which is in line with previous findings [19]. Brunelli et al. reported a significantly higher value of sTM in AAA patients associated with elevated homocysteine level, a factor alleged to contribute to endothelial injury. In the present study, sTM concentration maintained elevated in the subgroup of patients without clinical manifestations of atherosclerosis, which may suggest that an increased sTM is an independent feature of AAA rather than the effect of atherosclerotic alteration commonly occurring among AAA patients. However larger population-based studies are needed to confirm this hypothesis.

Some studies indicate the importance of the parameters of endothelial dysfunction in the monitoring of AAA presence and progression. Sung et al. reported an impairment of endothelial function in AAA patients reflected by a decreased value of flow-mediated vasodilatation (FMD) and a reduced number of endothelial progenitor cells (EPCs), which were correlated with a large AAA diameter [36]. Similar results were obtained by Parietti et al. who observed a strong inverse correlation between the EPCs levels and aneurysm diameter in 27 patients with thoracic aortic aneurysm [37]. However, the results of our study failed to show any association between sTM and the AAA severity, reflected by the aneurysm's size. An elevated concentration of sTM was observed in patients with small as well as large AAA diameter without any gradation influenced by the disease severity.

In our study we also observed the tendency of sTM to be increased most in patients qualified for EVAR repair. This phenomenon may be explained by the fact that EVAR is recommended for elderly patients with various coexisting diseases, which may act as additional factors disrupting endothelial layer integrity and influencing the sTM concentration in positive manner. We have also examined the effect of EVAR as well as open AAA surgery on postoperative sTM concentration. A significantly increased sTM concentration was noticed 48–96 h after surgical intervention but only in patients who had underwent OR. The OR is associated with ischemia-reperfusion (I/R) responsible for the extensive reactive oxygen species (ROS) generation and systemic inflammation [38–40]. Both ROS and inflammatory mediators may initiate the endothelial injury. Barry et al. demonstrated that venous blood taken during reperfusion stage of OR causes upregulation of endothelial cell ICAM-1 expression in vitro [41]. Endothelial cell membrane integrins that have been shed from the cell surface have also been demonstrated in the blood of AAA patients after surgical repair of AAA, with considerably high levels of soluble ICAM-1 in shocked AAA patients and in nonsurvivors of ruptured AAA [42]. Kokot et al., who have assessed the dynamics of endothelium injury markers measured during elective AAA surgery, reported the highest rise of VCAM-1 within 48 h after surgical procedure [43]. The results of the present study indicate that sTM, similarly like VCAM-1 described above, may be a useful marker in monitoring endothelial injury occurring in the early postoperative period after OR. Some investigators have found that EVAR is associated with a lower induction of proinflammatory cytokines and oxidative stress rather than the conventional technique [38, 44, 45]. This fact may explain no difference between baseline and postoperative value of sTM after EVAR observed in present study, which result from its less damaging impact towards endothelial layer. Although EVAR, when compared to OR procedure, seems to be less invasive and is associated with a reduced perioperative complications, in a long-term comparison no significant differences were observed in the total mortality or aneurysm-related mortality between these two techniques [46, 47].

The mechanisms initiating the vascular damage in AAA are still poorly understood. Inflammation, proteolysis, and oxidative stress are supposed to take part in the process of the destructive remodeling of the aorta in pathogenesis of AAA [20–22, 48–50]. In the present study we investigated the relationship between sTM and various clinical and biochemical parameters, especially those that could be involved in the endothelial damage. We demonstrated that an increase in sTM concentration shown in AAA patients is associated with an increased TAG level. Moreover, in the subgroup of AAA patients with the highest sTM concentration, the value of cholesterol and hsCRP levels was significantly increased. These findings confirm some previous studies pointing to close connection between lipid abnormalities, inflammation, and endothelial damage in AAA [51–56]. Due to the fact that the same factors participate in the atherosclerotic plaque formation, it is difficult to state whether the above observations represent a true association with AAAs or if they simply reflect the higher incidence of atherosclerotic disease found in this group of patients. Both diseases affect arteries, share predisposing risk factors, and exhibit similar immune and inflammatory cell infiltrates [57]. Moreover, one of the hypotheses assumes that AAA develops as a pathological response to aortic atherosclerosis [24]. Therefore, in the analysis of mechanisms underlying AAA development, the exclusion of atherosclerosis would be complicated and misleading. Numerous epidemiological studies indicate the decreased value of diastolic blood pressure is a strong marker of carotid atherosclerotic plaques [58–61]. A drop in diastolic BP associated with an increase in sTM concentration which was observed in the present study suggests that atherosclerosis may attenuate the breakdown of vascular integrity leading to the AAA formation.

Except for the atherosclerosis as a main factor involved in AAA development, some other alternative hypotheses have been postulated. Recently the role of oxidative stress in the pathogenesis of AAA has been increasingly investigated [21, 62, 63]. It is assumed that the overproduction of reactive oxygen species (ROS) may subsequently induce inflammation, matrix metalloproteinase (MMP) activities, and smooth muscle cell apoptosis (i.e., the processes enhancing pathological artery wall remodeling). It is well known that ROS can react with different major components of tissues such as lipids, proteins, and DNA causing their oxidative modification and formation of products which serve as an important indicator of redox status. The interaction of ROS with proteins leads to the formation of advanced oxidation protein products (AOPPs) and advanced glycation end products (AGEs) which constitute novel markers that provide information on the degree of oxidative damage in organism [64, 65]. Although AOPPs and AGEs have been evaluated in several pathological conditions, including diabetes, inflammation, renal failure, and Alzheimer's disease, the knowledge about their concentrations in patients with aortic diseases, specifically aneurysms, is still limited [64–70]. In the present study the correlation of sTM with products of oxidative modification of proteins such as carbonyl groups content, AOPP, and AGEs was investigated. We demonstrated a significant positive association between sTM and AGEs concentration. The highest value of AGEs was observed in patients who fell into the highest quartile of sTM concentration. This observation may confirm the negative effect of oxidative stress on the endothelial layer in AAA pathogenesis. However, it has to be noted that not only are AGEs a parameter reflecting the process of oxidative damage but they per se demonstrate a destructive potential towards endothelial cells [71]. AGE-mediated damage occurs via binding of AGEs to the receptor of advanced glycation end products (RAGE) located on vascular cells. The activation of RAGE by AGEs increases endothelial permeability to macromolecules and results in the alteration of the cell surface structure, from that of an anticoagulant to a procoagulant endothelium [72]. Moreover, the signals from the RAGE-ligand complex trigger an inflammatory cascade resulting in the increased liberation of cytokines, ROS, and proteases by the arterial wall [71, 72]. RAGE is expressed as both full-length membrane-bound forms and various soluble forms lacking the transmembrane domain (soluble RAGE: sRAGE). sRAGE is able to act as a “decoy/scavenger receptor,” by binding proinflammatory ligands and restraining them from binding with membrane-bound RAGE [71–73]. In our study we demonstrated an inverse relation between sTM and sRAGE concentration. It may suggest that in patients with low concentration of sRAGE the endothelial damage is more severe due to insufficient protection from ligands such as AGEs which easily bind with RAGE receptors inducing reactions described above. It is worth noticing that the same positive correlation observed between sTM and AGEs was not found in case of other studied markers of oxidative stress (AOPP, carbonyl groups). This may reflect not the negative effect of an increased systemic oxidative stress on endothelial layer but rather a destructive potential of AGEs being insufficiently inactivated by sRAGE. However, further experiments would be required to test this hypothesis.

## 5. Conclusion

The results of the present study indicate that AAA is associated with endothelial damage reflected by an increased concentration of sTM. However its level did not distinguish between small and large aneurysm. Among the various factors that have been studied in our research, enhanced oxidative stress, inflammation, and lipid abnormalities seem to be implicated in the process of vascular damage. The comparison between two surgical treatments of AAA revealed that conventional open repair may be associated with the more severe endothelial injury in the early postoperative period.

## Figures and Tables

**Table 1:** Clinical characteristics of the AAA patients and control group.

Parameter	Control (*n* = 30) Number (%)	AAA patients (*n* = 41) Number (%)
Age > 80 years	0 (0)	5 (12)
Gender (male/female)	20/10	32/9
Current smoker	7 (23)	20 (50)
Hypertension	2 (10)	30 (73)
Hypercholesterolemia	0 (0)	15 (38)
Coronary artery disease	0 (0)	17 (41)
Peripheral artery disease	0 (0)	18 (44)
Previous myocardial infarction	0 (0)	10 (25)
Cerebrovascular accident	0 (0)	3 (7)
Type 2 diabetes	0 (0)	11 (28)
Renal insufficiency	0 (0)	2 (6)
Pulmonary disease	0 (0)	3 (7)

**Table 2:** Anatomic inclusion for EVAR.

AAA diameter ≥ 5.5 cm
Proximal aortic neck length ≥ 15 mm
Proximal neck angel < 60°
External iliac artery diameter ≥ 7 mm
Absence of thrombi of extensive calcification in the proximal neck (>50% of the circumference)

**Table 3:** Criteria to be considered for high-surgical or anesthetic risk patients.

(A) Age ≥ 80 years
(B) Serum creatinine level ≥ 3 mg/dL
(C) Severe pulmonary dysfunction (defined as forced expiratory volume in first second < 1 L, PaO_2_ < 60 mmHg, PaCO_2_ > 45 mmHg, or small effort dyspnea)
(D) Severe cardiac dysfunction [defined as recent acute myocardial infarction (less than 3 months), ejection fraction of the left ventricle ≤ 25%, recent or recurrent symptomatic congestive heart failure (less than 3 months), severe and diffuse coronary artery disease (no anatomic conditions for revascularization) with or without unstable angina, symptomatic aortic stenosis, and unstable angina at rest.]

**Table 4:** Comparison between AAA patients categorized into quartiles according to sTM concentration.

	sTM (ng/mL)			
	Quartile I (sTM concentration < 2.19)	Quartiles II and III (sTM concentration 2.19–5.69)	Quartile IV (sTM concentration > 5.69)	*p* value
Age	72.14 ± 10.95	71.29 ± 9.96	72.57 ± 8.30	0.956^(b)^
BMI	27.64 ± 6.20	27.17 ± 3.63	30.61 ± 7.43	0.416^(b)^
Aneurysm diameter (mm)	66 (55–74)	66 (56–72)	59 (52–63)	0.610^(a)^
Systolic blood pressure (mm Hg)	138 (135–163)	135 (120–142)	125 (120–145)	0.230^(a)^
Diastolic blood pressure (mm Hg)	90 (82–90)	80 (80–85)	75 (59–83)	0.005^(a)^
WBC (10^9^/L)	7.84 ± 3.94	7.67 ± 2.31	7.99 ± 2.60	0.971^(b)^
RBC (10^12^/L)	4.51 (4.33–4.58)	4.58 (4.34–5.14)	4.98 (4.38–5.28)	0.259^(a)^
PLT (10^9^/L)	321 ± 186	218 ± 54	255 ± 61	0.122^(b)^
Fibrinogen (mg/dL)	295 (284–385)	280 (262–328)	418 (228–436)	0.330^(a)^
Uric acid (*µ*M)	346 (312–356)	363 (319–412)	367 (316–399)	0.518^(a)^
TAG (mM)	1.16 ± 0.44	1.30 ± 0.59	2.83 ± 1.70	0.023^(b)^
Cholesterol (mM)	4.36 (3.23–4.81)	4.18 (3.13–4.43)	4.90 (4.62–4.97)	0.029^(a)^
LDL-cholesterol (mM)	2.40 (1.15–3.30)	2.15 (1.47–2.90)	2,30 (1.15–3.00)	0.566^(a)^
HDL-cholesterol (mM)	1.61 ± 0.69	1.22 ± 0.40	1.21 ± 0.24	0.079^(b)^
hsCRP (mg/L)	12.03 (6.87–15.53)	5.64 (1.25–11.00)	16.72 (7.72–23.26)	0.031^(a)^
AOPP (*µ*M/mg protein)	6.14 (4.06–8.03)	4.10 (2.74–6.60)	4.74 (3.83–6.08)	0.294^(a)^
AGEs (*µ*g/mg protein)	0.17 (0.11–0.19)	0.22 (0.11–0.19)	0.23 (0.16–0.46)	0.042^(a)^
RAGE (pg/mg protein)	1.81 (0.86–2.01)	0.75 (0.41–1.48)	0.71 (0.59–1.63)	0.030^(a)^
Carbonyl groups (nM/mg protein)	0.88 (0.70–1.34)	0.93 (0.71–1.00)	1.03 (0.66–1.98)	0.532^(a)^

^(a)^Results shown as median and interquartile range, comparison done by using Kruskal-Wallis test; ^(b)^results shown as mean ± standard deviation, comparison done by using one-way ANOVA test.

BMI: body mass index, WBC: white blood cells, RBC: red blood cells, PLT: blood platelets, TAG: triglycerides, hsCRP: highly sensitive C-reactive protein, AOPP: advanced oxidation protein products, AGEs: advanced glycation end products, and sRAGE: soluble receptors for advanced glycation end products.
